# Recent Advances in Additive Manufacturing and 3D Bioprinting for Organs-On-A-Chip and Microphysiological Systems

**DOI:** 10.3389/fbioe.2022.837087

**Published:** 2022-02-17

**Authors:** Mario Rothbauer, Christoph Eilenberger, Sarah Spitz, Barbara E. M. Bachmann, Sebastian R. A. Kratz, Eva I. Reihs, Reinhard Windhager, Stefan Toegel, Peter Ertl

**Affiliations:** ^1^ Karl Chiari Lab for Orthopaedic Biology, Department of Orthopedics and Trauma Surgery, Medical University of Vienna, Vienna, Austria; ^2^ Institute of Applied Synthetic Chemistry, Vienna University of Technology, Vienna, Austria; ^3^ Austrian Cluster for Tissue Regeneration, Vienna, Austria; ^4^ Ludwig Boltzmann Institute for Arthritis and Rehabilitation, Vienna, Austria; ^5^ Ludwig Boltzmann Institute for Experimental and Clinical Traumatology in the AUVA Research Centre, Vienna, Austria; ^6^ Institute of Chemical Technologies and Analytics, Vienna University of Technology, Vienna, Austria; ^7^ Ludwig Boltzmann Research Group Senescence and Healing of Wounds, Vienna, Austria; ^8^ Division of Orthopedics, Department of Orthopedics and Trauma Surgery, Medical University of Vienna, Vienna, Austria

**Keywords:** bioprinting application, organs- and tissues-on-a-chip, Microphysiological systems, STL, 2PP, droplets

## Abstract

The re-creation of physiological cellular microenvironments that truly resemble complex *in vivo* architectures is the key aspect in the development of advanced *in vitro* organotypic tissue constructs. Among others, organ-on-a-chip technology has been increasingly used in recent years to create improved models for organs and tissues in human health and disease, because of its ability to provide spatio-temporal control over soluble cues, biophysical signals and biomechanical forces necessary to maintain proper organotypic functions. While media supply and waste removal are controlled by microfluidic channel by a network the formation of tissue-like architectures in designated micro-structured hydrogel compartments is commonly achieved by cellular self-assembly and intrinsic biological reorganization mechanisms. The recent combination of organ-on-a-chip technology with three-dimensional (3D) bioprinting and additive manufacturing techniques allows for an unprecedented control over tissue structures with the ability to also generate anisotropic constructs as often seen in *in vivo* tissue architectures. This review highlights progress made in bioprinting applications for organ-on-a-chip technology, and discusses synergies and limitations between organ-on-a-chip technology and 3D bioprinting in the creation of next generation biomimetic *in vitro* tissue models.

## Introduction

The homeostasis and proper function of complex living biological structures such as organs and tissues are guided the interplay between cell communication, tissue composition and architecture governs development, repair and (dys)function. For instance, direct and indirect communication of individual cells and cell populations by means of cell-to-cell contact between similar or different phenotypes and soluble cell signalling molecules (Rothbauer et al., 2018) are associated with proper tissue function. It is important to note that soluble indirect communication plays not only important role in tissue homeostasis but is also involved in many pathological processes such as trauma, inflammation, allergic responses and arthritis (Dinarello, 2000; Kupper and Fuhlbrigge, 2004; McInnes and Schett, 2007; Gould and Sutton, 2008; Eming et al., 2014). Both direct and indirect cell communication processes have been successfully modelled using a variety *in vitro* cultivation techniques including single-cell cultures (e.g. microcavities, cell traps), and two-dimensional monolayer cultures (e.g. multi-well plates and Transwell membrane systems) as well as microfluidic and lab-on-a-chip systems (Kratz et al., 2019b) that can separate, control and manipulate direct and indirect cell communication of various heterogenous cell types in more precise manner (De Windt et al., 2015; Ellison et al., 2016; Bachmann et al., 2018; Zirath et al., 2018; Rothbauer et al., 2019). More recently, advanced microfluidic tissue-like models also called microphysiological/organ-on-a-chip systems have shown the ability to control both intra and inter-cellular communication processes as well as providing necessary architectural features seen in living tissue and organs (Ergir et al., 2018; Kratz et al., 2019b). For instance, a variety of advanced three-dimensional (3D) cell culture techniques have been implemented in organ-on-a-chip systems such as hanging drop spheroids, hydrogel microtissues und matrix-free self-assembled organoids inside microfluidic channel networks to precisely control the cellular microenvironment with high temporal and spatial resolution (Ling et al., 2007; Frey et al., 2014; Yu et al., 2019). Organ-on-a-chip technology can, in principle, recapitulate a number of key aspects of any human tissue and organ function as demonstrated for lung, liver, kidney, skin, eye, musculoskeletal tissues and many more as well as heart (Rothbauer et al., 2015; Rothbauer and Ertl, 2018; Kratz et al., 2019b; Rothbauer et al., 2021).

Despite recent advances in generating complex cell and tissue models inside microfluidics and organs-on-a-chips a major challenge still remains and is associated with the establishment of defined two- and three-dimensional spatially-resolved individual cell layers needed to form complex heterogenous architectural features of human tissue. Although techniques to control the spatial context of cells have been available for decades involving two-dimensional (2D) cell patterning using metal biomolecule and protein patterns (Khademhosseini et al., 2003) as well as smart switchable biointerfaces (Yousaf et al., 2001), their application in organ-on-a-chip technology is still in its infancy. Only recently, a variety of microfluidic techniques to create patterns of proteins, cells, and three-dimensional (3D) cell populations have been introduced including microcontact printing, microfluidic patterning, laminar flow patterning and more advanced techniques employing moveable actuators, anisotropic surfaces, micropillars and microcavities to control cell-laden hydrogel compartments (Rothbauer et al., 2015). The most recent addition to the toolkit to create spatially defined cultures of human cells that resemble tissue architecture is additive manufacturing using cell-laden bioinks as well as blank hydrogel templates that are consecutively populated with cells due to improvement in and affordability of bioprinters (Kahl et al., 2019). Even though industrial 3D printing goes back to stereolithography introduced in the 80s, it took the scientific community decades to translate additive manufacturing techniques including stereolithography, extrusion printing, inkjet printing, laser-assisted printing and two-photon polymerization to the biological fields of tissue engineering, biomedical engineering and disease modelling. The main drawbacks of any additive manufacturing approach in the life sciences are the invasiveness of the printing techniques (i.e., biocompatibility and cytotoxicity) and the still limited availability of suitable organic and inorganic functional bioinks. In other words, all printing techniques are known to induce cell disruption due to high shear stress during printing for droplets and nozzles, thermal load or phototoxicity for laser-based techniques. Also, more artificial polymers such as poly (ethylene glycol)dimethacrylate (PEGDMA) are used to replace approaches based on gelatin, collagens, basement membrane extracts, and blood-derived fibrin components (Heid and Boccaccini, 2020). It is important to highlight that the type of extracellular matrix used as bioinks severely impacts the (patho)physiological quality and biological relevance of the printed tissue construct. For instance, deviating matrix densities, porosities and compositions of e.g. collagen can cause a healthy organ tissue to become fibrotic due to the presence of pathological or pathogenic ECM components (Verrecchia and Mauviel, 2007; Wynn, 2008). However, blending of bioinks with hydroxyapatite or bioactive glass can create bioactive compostive materials with a stiff osteogenic niche (Heid and Boccaccini, 2020). Another more technical limitation is associated with the successfully integration of 3D printed tissue structures in microfluidic devices, which commonly comprise of tightly sealed and enclosed microfabricated structures that prevent effective polymerization along channel walls. In other words, the combination of 3D bioprinting and microfluidics technology is limited by the compatibility and adaption of bioprinting techniques for most frequently closed microsystems ranging from a few micrometers up to millimeters.

To address the above issues, this review focuses on recent progress in additive manufacturing for organ-on-a-chip and microphysiological systems, representing the current most advanced 3D culture techniques for human cells and tissue-engineered constructs *in vitro*. The first section provides a broad technological summary of the basic working principles behind additive manufacturing techniques. The second part of the review highlights how additive manufacturing has been applied to these intricate microfluidic models over the last 5 years, resulting in more refined 3D cell models that resemble human tissue not only on a functional but also on an architectural level. Therefore, the presented literature focuses on recent 3D-bioprinting advances for the fabrication of organ-a-on-a-chip systems involving living human organ- or tissue constructs. Additionally, laser-assisted and light-assisted printing techniques are discussed for potential organ-on-a-chip applications, even though it has not been used for microfluidic models that resemble human physiology to date. Finally, additive manufacturing techniques for selected future applications in organ-on-a-chip technology are examined, and a possible outlook for future requirements is given based on the current state-of-the-art in bioprinting and *in vitro* bioengineering.

### A Summary on Additive Manufacturing Technologies for Bioprinting Applications

**FIGURE 1:**
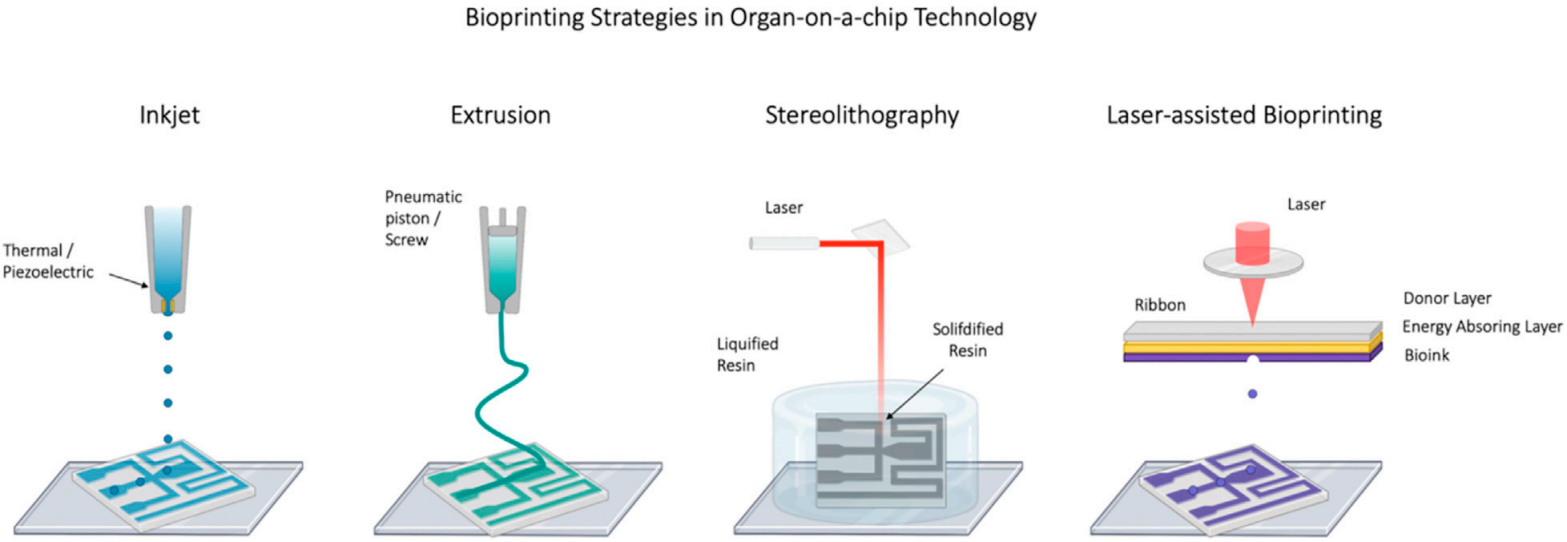
Schematic representation of bioprinting technologies including inkjet, extrusion (FDM), stereolithography (SLA) and laser-assisted bioprinting strategies. Created with Biorender.com.

A schematic overview of current additive manufacturing techniques for organ-on-a-chip applications is illustrated in Figure 1 and outlines inkjet printing, extrusion bioprinting, laser-assisted printing as well as stereolithography and two-photon polymerization. Inkjet bioprinting exploits either thermal, piezo-electric or electromagnetic forces to expel droplets of bioink onto a substrate positioned on top of an electronically controlled stage (Mohebi and Evans, 2002; Cui et al., 2012a). Despite of the high temperature maxima of up to 300°C developing within the printer’s nozzle, localized heating of the bioink is restricted to short time scales in the range of a few microseconds, enabling the deposition of cells without significant loss in cellular viability (Xu et al., 2006; Cui et al., 2010). This non-contact printing technique displays several advantages including high speed and broad availability as well as low acquisition costs and has therefore been widely employed in the printing of cartilage, bone, skin and vascular constructs (Cui et al., 2012b; Skardal et al., 2012; Lee et al., 2014; Wang et al., 2017). However, low precision, nozzle clogging, mechanical and thermal cell distortion/disruption, but most importantly the requirement of low viscosity inks (3.5–12 mPa.s) and therefore low cell concentrations have limited the applicability of this strategy for many biological implementations (Bishop et al., 2017; Zhang et al., 2019).

Similar to the inkjet technology, extrusion bioprinters employ either mechanical or pneumatic forces to extrude bioink in a continuous cylindrical stream through a nozzle onto a substrate (Zhang et al., 2019). Extrusion bioprinting constitutes the most common academic as well as commercialized bioprinting strategy today and is characterized by its ability to deposit materials of higher viscosities, such as bioinks, at physiologically relevant cell densities (Murphy and Atala, 2014). As such, extrusion bioprinting has been used for the recapitulation of tissue types of high cell density including aortic valve conduits and vascular grafts (Norotte et al., 2009; Duan et al., 2013). Disadvantages of the technique include low cell viability as well as distorted cellular structures as a result of the high pressure exerted onto the cells (Chang et al., 2008). More recently, extrusion bioprinting has been adapted to microfluidic applications by the design of coaxial nozzles, that enable the formation of microchannels as well as vascular network structures (Gao et al., 2015; Jia et al., 2016).

In stereolithography (SLA), UV light or a laser is directed in a specific pattern over a liquid photopolymerizable polymer, resulting in the curing of the polymer and the formation of 3D structures. Patterning of the printed structure is performed using digital micromirror arrays, enabling structures with a high intrinsic complexity, flexibility, and scalability. While stereolithography is similar to laser assisted bioprinting it uses a layer by layer process, resulting in higher speed at simultaneously improved resolutions of <25 μm (Guvendiren et al., 2016). Stereolithographic strategies allow the use of high accuracy polymers such as acrylic and epoxies, but are limited to relatively few materials and troubled by the need of intense UV irradiation as well as lengthy post processing times (Li et al., 2016). Because of this difficulty, SLA was not considered a good candidate for multi-material additive manufacturing until recently (Guzzi et al., 2021). However, in a recent publication, Khatri et al. present an approach for realizing a low-cost multi-material stereolithography 3D printing process for the fabrication of structural features in the 200–300 µm range with two different curable resins (Khatri et al., 2020).

Two-photon polymerization (2PP) is a specific form of stereolithography, enabling the fabrication of 3D constructs with sub-diffraction limit resolution. Consequently, 2PP technology is frequently used for the construction of microfluidic devices and provides the ability to replicate physiologic microenvironments *in vitro* (Lee et al., 2008). However, owing to its high resolution (<100 nm) 2PP has very lengthy fabrication times (>several hours) unsuitable for the construction of bigger tissue analogues. Furthermore, 2PP is still limited to the use of photosensitive polymers from the microelectronics industry, which generally display lower biocompatibility (Nguyen and Narayan, 2017). However, novel and improved commercial photo-sensitive bioinks compatible with 2PP are currently being developed to fill this technological gap (Dobos et al., 2020b).

In laser-assisted bioprinting, laser pulses are directed towards a so-called bioink-coated ribbon where the ribbon is supported either by a layer of gold or titanium, which acts as an energy transmitter. In this technique, the bioink is suspended on the bottom of the ribbon, gets vaporized by a directed laser beam, and is subsequently ejected onto the underlying substrate. Trough repetitive projection of bioink droplets, this process enables the formation of 3D structures of high resolution, near the scale of a single cells (∼10 μm) (Guillotin et al., 2010; Bishop et al., 2017). Owing to its high resolution, laser-assisted bioprinting has already been used for the printing of micropatterned peptides, DNA as well as cell arrays (Skardal and Atala, 2015). Furthermore, this non-contact and nozzle-free strategy was shown to result in cell-loaded droplets of high cellular density (>10^8^ ml^−1^), underlining its applicability for often physiologically required high cell densities (Guillotin et al., 2010). However, laser-assisted bioprinting is a time-consuming process (ribbon preparation), characterized by high spatial resolution, high retention of cellular phenotypes as well as good cell viabilities (Bishop et al., 2017; Foyt et al., 2018).

Recently, a new paradigm in photopolymer-based additive fabrication called volumetric bioprinting has been proposed by Shusteff and colleagues, enabling the fabrication of 3D geometries on a time scale of seconds (Shusteff et al., 2017). This processing speed is achieved by the superposition of patterned optical fields from ultrafast laser beams, projected at orthogonal directions into a photo-sensitive resin. A series of holograms are sequentially applied to produce the desired 3D structure within a photosensitive resin. Using this unique holographic patterning system, various 3D shapes made of polyethylene glycol diacrylate have been fabricated by a single light exposure of up to 10 s. These structures, however, were limited in their geometry due to the prismatic nature of the overlapping beams. Another novel approach, denoted as “computed axial lithography,” has been developed to overcome these limitations (Kelly et al., 2019). This technique is based on tomographic reconstruction, with mathematical optimization to generate a set of projections to define an arbitrary dose distribution within a target volume optically and to cure the entire volume simultaneously. Inspired by computed tomography, this printing process enables the production of more complex objects by using 2D dynamic light fields. Technical photopolymers such as acrylates and elastomeric resins have been printed, showing the ability to resolve features down to 80 µm (Bernal et al., 2019). These new techniques are opening novel ways to upscale the production of bioprinted constructs and their application in especially in tissue engineering, regenerative medicine, and soft robotics including also organs-on-a-chip. The selection criteria for bioinks and their properties depend on the specific application (*e.g.*, target tissue) and the type of cells as well as the general bioprinting method. Even though natural biomaterial bioinks are highly used (i.e., agarose, alginate, collagen, hyaluronic acid, fibrin, cellulose, silk, matrigel), ECM-based bioinks, cell aggregates as well as spheroids but also more synthetic materials (i.e., polylactic acid, polyethylene glycol, polycaprolactone) are also showing promising results towards the development of functional tissues or organs using 3D bioprinting technology (Gopinathan and Noh, 2018).

The printability of the bioink strongly depends on the different parameters such as viscosity of the solution, surface tension of the bioink, the ability to crosslink on its own, and surface properties of the printer nozzle. While natural polymers are more suitable to mimic extracellular matrix resulting in effective cell growth, synthetic polymers offer tailorable mechanical properties and printability (Gungor-Ozkerim et al., 2018). Moreover, the viscosity of the bioink formulation should be tunable to facilitate the usage of the same bioink in different commercially available printing machines. The droplet and inkjet-based printers require a solution viscosity of 10 mPa.s, whereas the extrusion-based involves a minimum of 30–6  ×  10^7^ mPa.s However, laser-assisted printing, requires a viscosity of 1–300 mPa.s (Hölzl et al., 2016; Ozbolat et al., 2016). Novel bioinks formulated from several materials at different scales and higher resolutions may allow overcoming past limitations. The development of innovative multi-material bioinks, brings the biomedical engineering community closer to the clinical expectations of fabricated constructs capable of replicating native tissues. For instance, vascularized tissues within 3D perfusion chips were co-printed using cell-laden, fugitive, and silicone inks. Kolesky et al. (2016) First, the silicone ink is printed on a glass substrate and cured to create customized perfusion chips. Next, the cell-laden and fugitive inks are printed on-chip and encapsulated with a castable ECM-based bioink containing gelatin, fibrinogen, cells, thrombin, and transglutaminase. This process yields a pervasive network of interconnected channels lined with HUVECs. The resulting vascularized tissues are perfused *via* their embedded vasculature on-chip over 6 weeks using an external pump to promote differentiation of human mesenchymal stem cells toward an osteogenic lineage *in situ*.

### Application of Additive Manufacturing Techniques in Organ-On-A-Chip Research

The following sections focus on specific bioprinting solutions with the aim of improving the functional and architectural capabilities of organ-on-a-chip systems, where subsections are arranged according to the application frequency starting with extrusion-based followed by inkjet, laser- and light-assisted on-chip bioprinting techniques.

### Extrusion-Based Bioprinting as Method of Choice for Organ-On-A-Chip Systems

**FIGURE 2:**
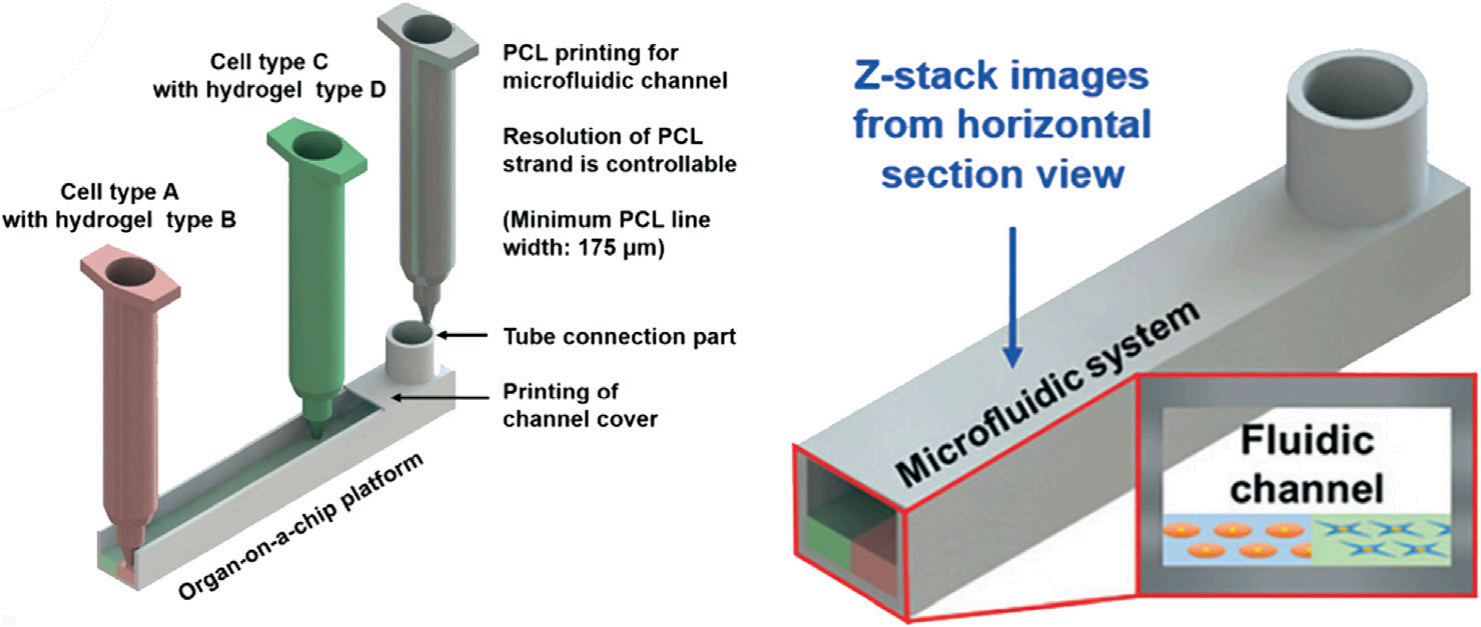
Holistic multi-material extrusion printing approach for a fully sealed liver-on-a-chip model printing both the microfluidic channel network as well as heterotypic 3D multi-cellular constructs. Reproduced from Lee et al. (Lee and Cho, 2016) with permission from the Royal Society of Chemistry.

In extrusion bioprinting, hydrogels are extruded through a nozzle and deposited on a printing bed, which is currently the predominant method used for organ-on-a-chip applications. Here the broad availability of affordable bioprinters and usage of well-established hydrogel systems such as gelatin, collagen, alginic acid (alginate) or fibrin has fostered the early application of bioprinting in microfluidics. After extrusion, constructs are polymerized either by temperature elevation, enzymatic reaction, or UV-crosslinking, depending on the employed functional hydrogel systems. This, however, means that in applications with organ-on-a-chip system, the cell-laden hydrogels need to be printed into the microfluidic chip prior to sealing of the microfluidic chamber. Since the nozzle needs to deposit and structure cell material into open cavities, secondary device assembly and stability remains a technological challenge. One of the most holistic multi-material printing approaches to meet this challenge is the simultaneous printing of the microfluidic device in parallel to the hydrogel-based tissue constructs, as shown by Lee et al. (Lee and Cho, 2016) in Figure 2. A sophisticated one-step biofabrication pressure extrusion process was employed to manufacture a liver-on-a-chip system using a poly (ε-caprolactone) microfluidic device and temperature-polymerizing gelatin and collagen type I hydrogels with embedded HEPG2 liver and HUVEC vascular cells. The liver-on-chip device featured a 400 µm layer of hydrogel-embedded HEPG2 cells beneath a single-cell endothelial cell layer. The generated fluidic channel successfully emulated organotypic functions such as albumin and urea secretion superior to hepatocyte mono-cultures as well as off-chip hepatocyte-endothelial co-culture. This study clearly demonstrated what the combination of bioprinting and organs-on-a-chip as holistic print-on-demand approach can accomplish. This approach holds great promise for future applications including the replacement of the frequently used silicon-based polymer polydimethylsiloxane (PDMS) biochips, which are not fit for industrial purpose due to unwanted material properties as small molecule absorption, protein adsorption, permeability to water vapor, material swelling and ageing as well as chemical sensitivity (Mukhopadhyay, 2007; Carter et al., 2020).

**FIGURE 3:**
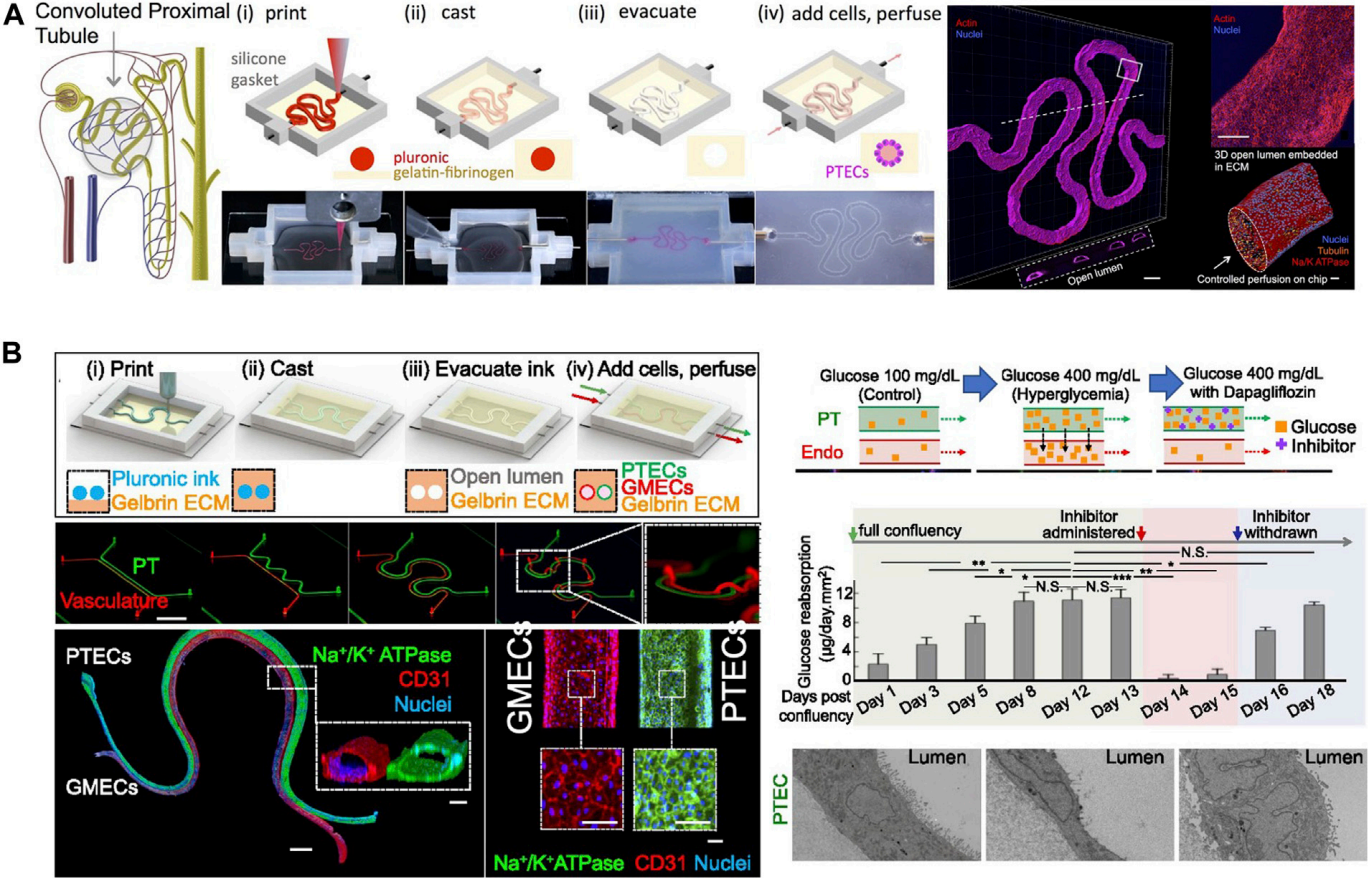
**(A)** Extrusion printing of a sacrificial tubular Pluronic construct and consecutive casting of Renal proximal tubule. Adpated from (Homan et al., 2016) with permissions. **(B)** follow-up study by Lin at el. used this principle to combine two lumenized structures within a kidney-on-a-chip system to study tubular vascular exchange. Adapted from (Lin et al., 2019) with permissions.

Nevertheless, most commercially available organ-on-a-chip systems that are pre-assembled have limited usability for extrusion bioprinting, since re-sealable devices using adhesives or gaskets are not amenable for high-volume production. As an example, Abudupataer et al. (Abudupataer et al., 2020) recently developed a device similar to Figure 2 to construct a biomimetic vessel-on-a-chip system. In contrast to the one-step process described above, the microfluidic layers were prefabricated from poly (methyl methacrylate (PMMA) using computer numerical controlled (CNC) engraving and sealed using adhesive tape after printing of the cell-laden hydrogels within the chambers. Here, GelMA hydrogel was used for printing of hydrogel-embedded human aortic endothelial cells on top of a smooth muscle cell (SMC) layer. This approach resulted in an optimized biomimicry of a smooth muscle phenotype, as demonstrated by upregulated αSMA and SM22 expression compared with the traditional culture system. Other, re-sealable devices consist of PDMS microfluidic chambers sandwiched between a more rigid substrate such as glass or PMMA that are used to compress the PDMS layers using a pressure-based approach to prevent leakage (e.g. compression by screws). The suitability of this approach to create small-scale bioreactors for perfusion of bioprinted tissues has been demonstrated by the Khademhosseini group for a liver-on-a-chip (Bhise et al., 2016) and a heart-on-a-chip platform (Yu Shrike Zhang et al., 2016). The liver-on-a-chip platform utilizes HepG2/C3A spheroids formed in PDMS microwells that are encapsulated within a GelMa hydrogel and printed into a resealable microfluidic device. Dynamic cultivation of 0.4 × 10^6^ cells preassembled into ∼175 µm wide spheroids within a 2 ml cultivation chamber enabling maintenance of the tissue constructs for 30 days and resulted in enhanced secretion rates of ceruloplasmin, A1AT, transferrin and albumin. Additionally, the heart-on-a-chip device is based on an off-chip bioprinting procedure with subsequent embedding of the scaffold into a resealable microfluidic organ-on-a-chip device. The scaffolds are printed by a sophisticated technique using a mixture of hydrogel precursors of alginate and GelMA, leading to formation of microfibers and customizable 3D deposition. For creation of an endothelialized myocardium, HUVEC endothelial cells were embedded during the bioprinting process and allowed to organize into a layer of confluent endothelium directly on the solid fiber surface before seeding of neonatal rat cardiomyocytes. These constructs were subsequently transferred to the microbioreactor after a cultivation time of 3 days and dynamically cultivated for up to 2 weeks demonstrating that an aspect ratio of 2 × 5 unit grids yielding soft elastic moduli showed most reliable long-term contraction amplitudes (*see* Figure 3A). Perfusion of the constructs significantly increased endothelial cell and cardiomyocyte viability and allowed for monitoring of cardiomyocyte beating frequency to demonstrate a dose-dependent response upon treatment with doxorubicin. A significant disadvantage of this system, however, is the lacking perfusability of the endothelial myocardium. Homan et al. (Homan et al., 2016), in turn, highlight the applicability of generating perfused renal proximal microtubules on chip. In contrast to classic build-up approaches, a convoluted renal proximal tubule was bioprinted using fugitive ink within a gelatin-fibrin hydrogel. After printing, the fugitive ink that acts as a sacrificial structure, was removed and the convoluted tubule was seeded with human proximal tubular cells that can be maintained within the device for longer than 2 months. The on-chip cultivation method coupled with perfusion of the proximal tubule resulted in enhanced epithelial properties, including a dose-dependent epithelial barrier disruption upon nephrotoxin introduction. As shown in Figure 3B, a follow up study by Lin et al. (Lin et al., 2019) used the same principle to combine two lumenized structures within a kidney-on-a-chip system to study tubular vascular exchange. The model featuring active reabsorption of solutes such as albumin and glucose was used to study the detrimental hyperglycemic effects on endothelial cell dysfunction, which could be recovered by incubation with glucose transport inhibitors. This study highlights how bioprinting can help to model tissue structure crosstalk, i.e., between epithelium and endothelium layers constituting architectural key principles found in many human tissue barriers.

### The New Kids on the Block: Inkjet Printing, Laser- And Light-Assisted Printing

**FIGURE 4:**
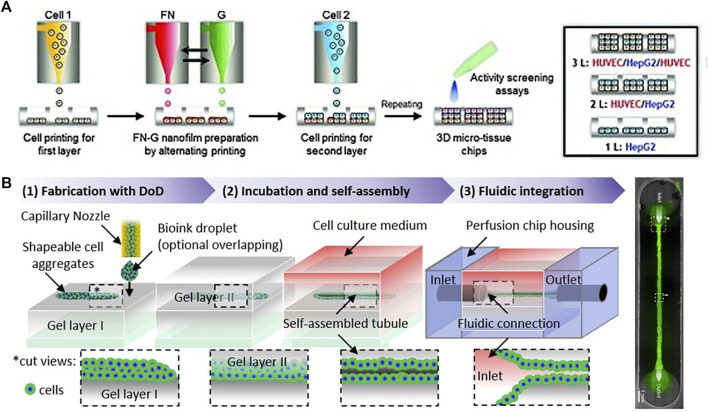
**(A)** Schematic illustration of the development of 3D hepatic-tissue structures by inkjet printing of single cells and proteins with layer-by-layer deposition. Adapted with permissions from Matsusaki et al. (Matsusaki et al., 2013) **(B)** Renal nephron-like tubular structures using a three-step layer-by-layer approach of printed cell aggregates sandwiched between two casted collagen I layers inside a microfluidic nephron-on-a-chip system. Adapted with permissions from Tröndle et al. (Tröndle et al., 2021).

Inkjet bioprinting is a contactless bioprinting method that precisely expels droplets of bioink onto a substrate positioned on top of an electronically controlled stage. Zhang et al. reported the first inkjet printing approach that was coupled with the creation of a microfluidic liver-on-a-chip device where HepG2 and U251 cells were encapsulated in sodium alginate and printed into arrays on glass slides covered by a PDMS layer with microfluidic channels (Jie Zhang et al., 2016). HepG2 cells and U251 cells were co-patterned in the microchip and used for drug metabolism and diffusion experiment. The prodrug tegafur was metabolized by HepG2 cells into the active anticancer drug 5-fluorouracil, leading to a growth-inhibiting gradient effect on U251 cells depending on the distance from the HepG2 cells. Similarly, Matsusaki et al. generated a chip containing multiple arrays of miniaturized human liver tissue by automatic inkjet printing as shown in Figure 4A (Matsusaki et al., 2013). The fabricated liver tissue chip consists of different cell layer compositions HepG2 cells and HUVECs, stacked with a fibronectin-gelatin film as a glue. The metabolic function and detoxification activity were most elevated in the triple-layered tissue, where the hepatocyte layer is sandwiched between an upper and lower endothelial cell layer. When treated with the hepatotoxic drug troglitazone (Rezulin), the triple-layered model exhibited the highest cytochrome P450 (CYP450)-mediated metabolism among the three models. Lee et al. also presented a multi-layer 3D liver fibrosis-on-a-chip system including hepatocytes, activated stellate cells, and endothelial cells using a cell-printing technique with gelatin bioinks (Lee et al., 2020). The developed disease model exhibited characteristics of liver fibrosis such as collagen accumulation, cell apoptosis, and reduced liver functions. Overall, inkjet printing improves the resolution of the cell droplets over the micro-extrusion printing method but cannot print large-scale biological structures with homogenous surface properties (i.e., several mms to cms). Despite its disadvantages, inkjet printing as shown in Figure 4B is favored for replicating narrow complex biological structures, because it offers high-resolution droplet printing (∼20 μm). Based on spheroidal self-assembly and lumen generation Tröndle et al. (Tröndle et al., 2021) reported inkjet printing of cell aggregates to fabricate a perfused tubular structure using a layer-by-layer approach similar to the renal model by Homan and colleagues mentioned in the previous section on extrusion-based systems (see also Figures 3A,B). Obviously the application potential is similar to extrusion bioprinting, however, regarding the practical limitations such as the requirement of low viscosity bioinks and the inherent inability to perform continuous flow, constructing 3D architectures is very challenging and therefore this method has been less applied to 3D bioprinting (Peng et al., 2018). Here, a multi-step procedure as shown in Figure 4B is necessary to first print the tissue construct within open channels and finally seal the microchamber, which is a strategy of limited capacity for industrial scale-up applications. A recent in-house-built 3D cell printing system also enables the combination of inkjet and extrusion-based modules for engineering of a diseased human skin (Kim et al., 2021). A dermal compartment was deposited using an extrusion-based module on a polycaprolactone extruded transwell system necessary for air-liquid interface culture of human epidermal keratinocytes. An inkjet-based module was applied following these steps to distribute epidermal cells as a monolayer. The printed skin structure was submerged for 3 days and exposed to the air-liquid interface for 7 days. The wounded skin model was formed by assembling a printing needle (16 gauge) in the printing head to create a small wound with 2 mm thickness in the skin model. For adding a perfusable vascular system, a coaxial cell printing technique was used and connected to a peristaltic pump.

**FIGURE 5:**
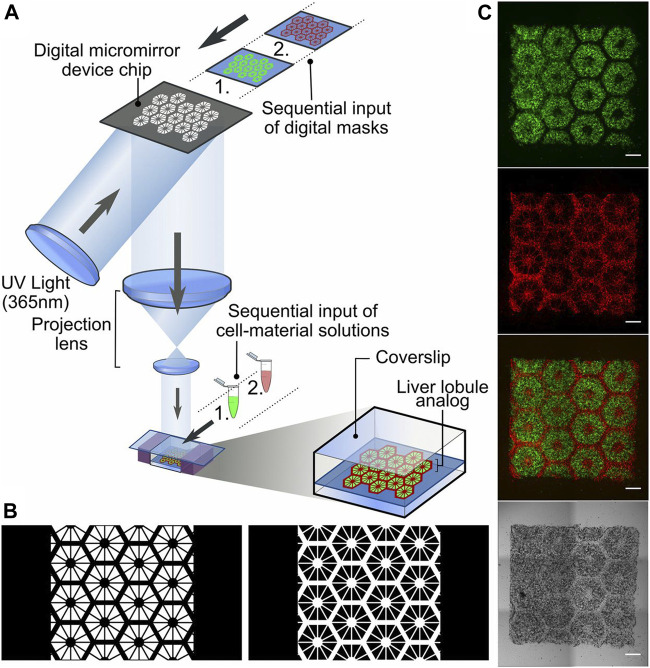
**(A)** Schematic diagram of a two-step 3D bioprinting approach with DLP. **(B)** Grayscale digital masks corresponding to polymerizing lobule structure (Left) and vascular structure (Right) designed for two-step bioprinting and **(C)** fluorescently labeled hiPSC-HPCs (green) in 5% (wt/vol) GelMA and supporting cells (red) in 2.5% (wt/vol) GelMA with 1% GMHA on day 0. (Scale bars, 500 µm). Reproduced with permissions from (Ma et al., 2016).

A laser-assisted printing approach overcomes some of the limitations of micro-extrusion and inkjet printing, which would offer highest droplet resolution due to the accuracy of laser targeting. Researchers have already managed to achieve resolutions needed to generate single-cell droplets (Bishop et al., 2017) and to print tissue constructs mimicking the 3D architecture of human bone (Catros et al., 2011) and skin (Koch et al., 2012; Sorkio et al., 2018). However, to date, no 3D-tissue construct was generated by laser-assisted bioprinting on a microfluidic platform yet. This may be attributed to the heat stemming from the laser that can adversely affect the cellular viability and the time-consuming process of producing the ribbon (Sears et al., 2016). Apart from laser assisted printing, another optical-based technique, commonly known as light-assisted printing or digital-light processing (DLP) is of great interest by using light interaction with the subject ink to either polymerize a photo-curable ink or help the deposition of the ink from a donor plane onto a substrate. As shown in Figures 5A–C Ma and colleagues fabricated liver lobule structures by DLP as shown in Figure 5, generating a 3D printed hexagonal anatomical feature of the lobule composed of various parts including a parenchymal tissue part of the human-induced pluripotent stem cell-derived hepatic progenitor cells (hiPSCs-HPCs) and the non-parenchymal tissue part with radial structure of the supporting cells (Ma et al., 2016). For this application, two bioinks were chosen containing 5% GelMA (∼5 kPa compressive stiffness similar to liver tissue) for the parenchymal tissue formation and the 25% GelMA/1% glycidal methacrylate-hyaluronic acid (GMHA; ∼4 kPa compressive stiffness) for vascularization. They found that this complex improved morphological organization, higher liver-specific gene expression levels, increased metabolic product secretion, and enhanced cytochrome P450 induction in comparison to 2D monolayer culture and a 3D HPC-only model. Moreover, the projection optics of the system focuses light patterns at micrometer-level resolution, thus enabling the biofabrication of the liver lobule hydrogel construct within several seconds with minimal UV illumination.

**FIGURE 6:**
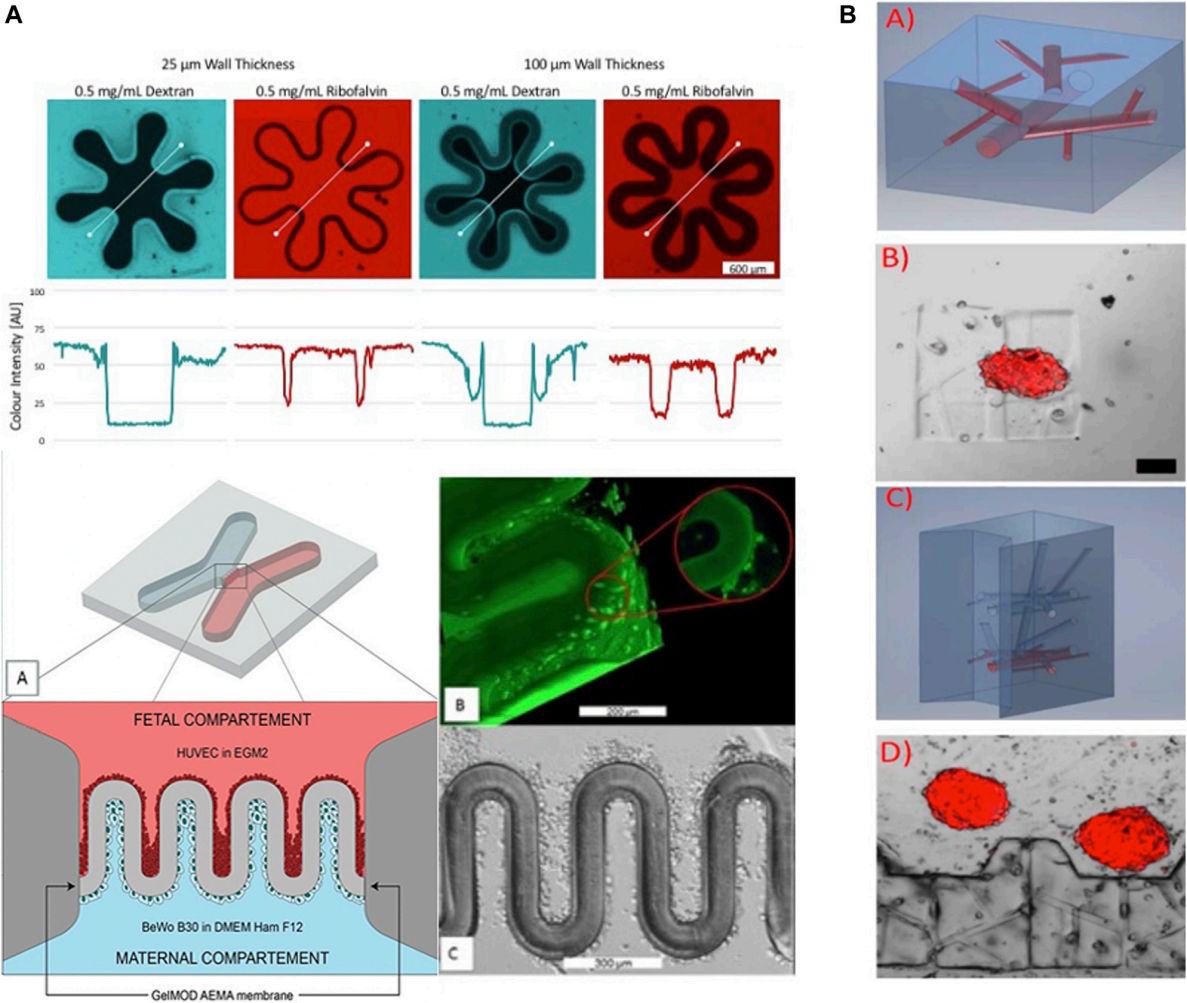
2PP on-chip bioprinting approaches for sub-micron resolution fabrication of **(A)** placental trophoblast barriers and **(B)** vascularized on-chip organoids. Adapted with permissions from Mandt et al. (Mandt et al., 2018) and Dobos et al. (Dobos et al., 2020a).

Stereolithography and two-photon polymerization are both optical techniques that, in principle, outperform extrusion and inkjet printing in terms of resolution and degree of freedom of the printed architecture using UV light or a laser to cure liquid photopolymerizable polymer. Zhang et al. used stereolithography to print hydrogels for vasculature-on-a-chip with biofunctionalized complex 3D perfusion networks (Zhang and Larsen, 2017). For instance, perfusable and mechanically stable hydrogel structures in self-containing chips featuring vascular-like networks were printed at high-resolution from poly (ethylene glycol) diacrylate (PEGDA, MW 700) hydrogel. This vascular system with a cross-section down to 100 μm × 100 µm was steadily perfusable for more native tissue-like dynamic culture of human umbilical-vein endothelial cells (HUVEC) after 7 days post-seeding. Furthermore, a co-culture of HUVEC with fibroblasts was established within the device, where the fibroblasts acted as support cells to improve the vascular microchannel system. Nonetheless, Miller and coworkers (Grigoryan et al., 2019) demonstrated based on SLA how to fabricate more biomimetic and authentic 3D approaches for vascularized systems (i.e., microfluidic vascular implants and valves) to improve the authenticity of lung, liver and cardiac microsystems also in the organ-on-a-chip sector. A 3D printed microfluidic perfusion device for multicellular spheroid cultures was established by Ong et al. (Ong et al., 2017) using SLA capable of direct immobilization and maintaining the viability and functionality of 3D multicellular spheroids by microstructures in the scale of 100 µm. Through pump-free gravity driven flow liver spheroids (HepG2) with a diameter of 130 µm were cultured for 3 days. Another high-resolution biomimetic approach was demonstrated by Mandt et al. as illustrated in Figure 6A with a biomimetic placental barrier structures were printed by 2PP bioprinting with in a microfluidic device (Mandt et al., 2018). Through printing photoactive methacrylates (GelMOD-AEMA) in villous membrane structures of 1,000 μm × 250 µm in a x-shaped poly-(ethylene glycol)-dimethacrylate (PEGdma, MW 700) chip the *in vivo* structure can be remodeled. A co-culture of HUVEC and human trophoblast choriocarcinoma cells (BeWo B30) were cultivated on each side of the printed membrane structures to remodel the barrier between fetal endothelial and maternal trophoblast side. Similarly, Dobos et al. demonstrated in Figure 6C that the capabilities of 2PP may even outperform SLA for bioprinting of highly branched vascular networks Figure 6B. Here vascular networks based on a thiol-ene hydrogel (Gel-NB-Gel-SH thiol-ene hydrogels) consisting of thiolated gelatin (Gel-SH) and gelatin-norbornene (Gel-NB) were established in the presence of endothelial cell spheroids and adipose-derived mesenchymal stem cells (AdMSC) support cells. Overall, 2PP bioprinting shows greatest promise for organ-on-a-chip applications, because this technique enables sub-micron resolution and the addition of a high degree of functional properties (i.e., stiffness, density and structural thickness) needed to manipulate not only cell-cell interactions but also fine-tune molecular and biomechanical gradients (Mandt et al., 2018; Zigon-Branc et al., 2019). Moreover, with the continuous introduction of improved printing hardware also speed and printing area is recently being optimized to improve the throughput of 2PP bioprinting (Weisgrab et al., 2020). Nonetheless, it has to be noted that 2PP printers are the least affordable systems creating a simple but strong economic and budgetary bias for broader life science applications especially in academia.

## Discussions on the Current State-Of-The-Art

Recently different bioprinting techniques have been introduced to enable three-dimensional control over *in vitro* organ models. Compared to bioreactors that can improve production of tissue-like structures, organs-on-a-chip constitute an enabling tool for multi-parametric monitoring of tissue-like structures. However, the translation of bioprinting technologies to microfluidic organ-on-a-chip systems is still in its infancy. This is reflected in the recent publications highlighted in the current mini review where the extrusion-based printing (six articles) is being most frequently used for organ-on-a-chip applications followed by inkjet printing (six articles), stereolithography (three articles) and two-photon printing (two articles) in the last 5 years. A general overview and summary of bioprinting techniques for organ-on-a-chip applications shown in Table 1 indicates that this technology distribution can be mainly explained by the initial investment costs associated with individual bioprinters, which is indirectly proportional to the number of publications. While extrusion-based printers can easily be built in a “do it yourself (DIY)” manner using syringes and automated stages amounting to a few hundred to thousand Euros/Dollars in component costs, the parts needed for building platforms for optical bioprinting techniques such as stereolithography and two-photon polymerization cost multiple tenth of thousands of Euros/Dollars. Similarly, available commercial systems can be found up to mid hundreds of thousand range. In addition to the equipment costs, another reason limiting the application of bioprinting methods to organ-on-a-chip application is associated with technological compatibilities. Although organs-on-a-chip microfabrication principles and bioprinting techniques have been well established individually over the last decades their combination and merging of these two fields is still a substantial challenge in itself. As an example, affordable and often bulky microfluidic chips based on glass and PDMS slides of several millimeters of height are not suited to interface with an extruder nozzle design or high-resolution objective of a two-photon setup. Even though extrusion and droplet-based methods seem more affordable and easier on the entry level for beginners of bioprinting, the major microfluidic problem is concerned with proper and long-term sealing stability and interfacing thereof as these systems need to be sealed after the printing process. Although some successes have been reported using pressurized manifolds and biomedical adhesives, a reliable organ-on-a-chip bonding strategy with 100% yield for long-term applications that enable bioprinting still has to be investigated especially for actuated systems similar to the lung- or gut-on-a-chip approaches (Kratz et al., 2019a). Additionally, all bioprinting technologies exert either mechanical and thermal strain as well as interface shear on the biological construct resulting in mechanical disruption and damage of cells leading to lower viability and high stress environments. Among bioprinting techniques, SLA and two-photon polymerization seem to be most compatible techniques with microfluidics technology, since they are capable of printing structures within tightly sealed compartments of microfluidic organs-on-a-chip. However, the optical properties of the material on the bottom or top of the printing compartment need to be carefully considered in order to ensure high transparency and adjustment of the refractive indices of material layers to enable high resolution bioprinting *in situ*. Another aspect that needs consideration is the inherent toxicity caused by radical generation by the photoinitiator-based photosensitive bioinks. It is important to note, however, that less toxic chemicals and formulations are recently introduced by photo-chemists to the photo-bioprinting community (Chen et al., 2018; Gopinathan and Noh, 2018). To overcome some of these material challenges, PDMS as the most frequently used material for biochip and organ-on-a-chip applications can potentially replace less biocompatible and established ink materials for extrusion based printing to generate gas-permeable and flexible biochips (Hinton et al., 2016).

**TABLE 1:** Overview of (bio)printing methods for organ-on-a-chip applications.

Bioprinting method	Resolution	Materials	Cell viability issues	Costs	Other remarks
Inkjet/Droplet	Single cells	Photo-sensitive polymers and bioinks	Toxicity of the photoinitiators; Mechanical shear; Surface impact	++/+++ (50–250 k€)	Post-printing seal necessary
Extrusion	>100 µm (depends on nozzle diameter)	Polymers and bioinks (±photoinitiators)	Extrusion shear	++ (>10 k€)	Post-printing seal necessary
SLA/DLP	∼50 µm	Photo-sensitive polymers and bioinks	Toxicity of the photoinitiators	+ (0.3–1 k€)	
Two photon polymerization (2PP)	<1 µm	Photo-sensitive polymers and bioinks	Toxicity of the photoinitiators	+++ (∼500 k€)	Thin glass substrates on one surface; optimization of refractive indices
Holographic	∼0.5 µm	Photo-sensitive polymers and bioinks	Toxicity of the photoinitiators	++++ (>1 M €)	Thin glass substrates on one surface; optimization of refractive indices of

**FIGURE 7:**
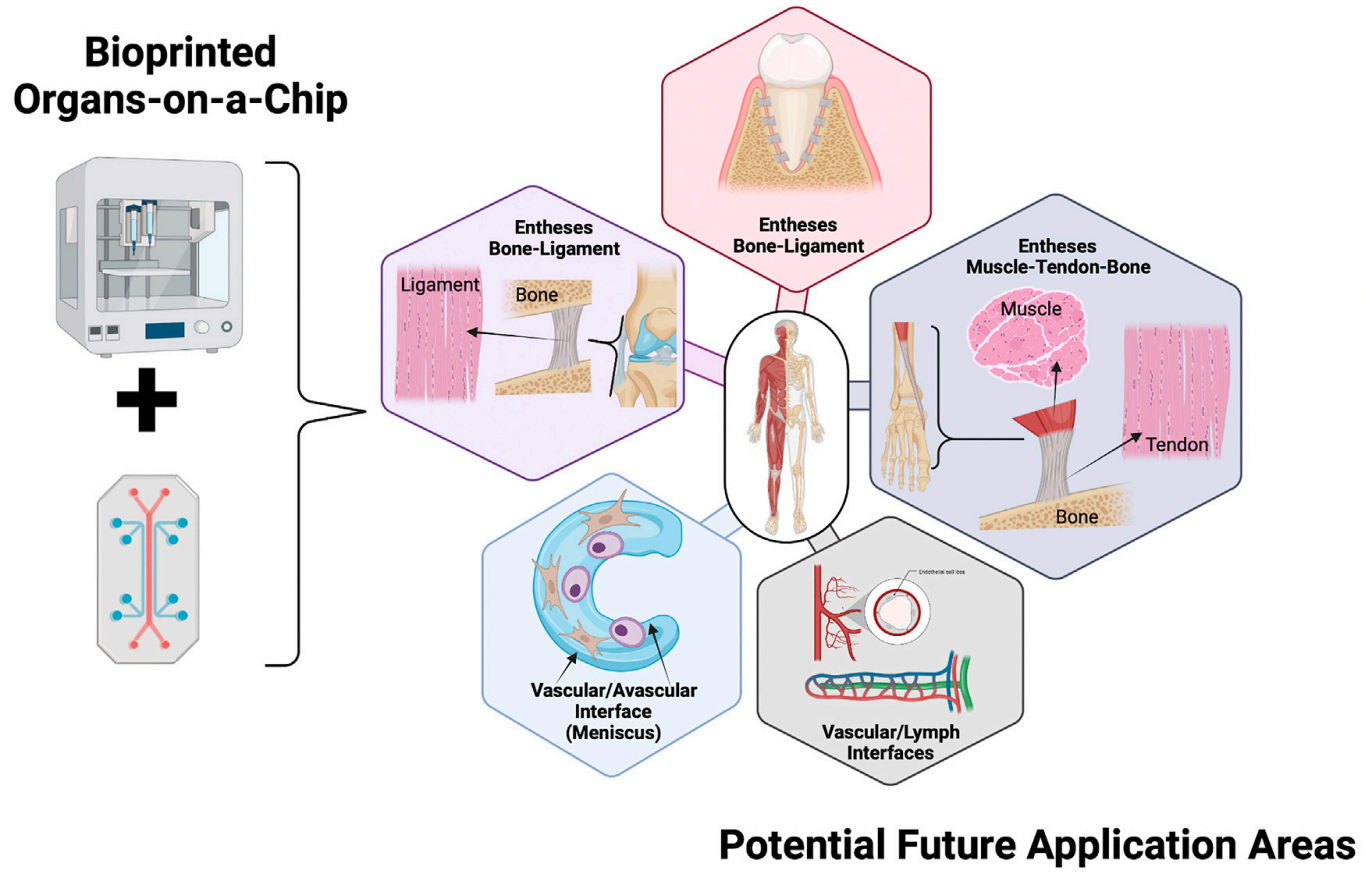
Schematic representation of the most challenging research areas for bioprinted multi-tissue organs-on-a-chip to become enabling technology. Created with Biorender.com.

Weighing the advantages of bioprinted organ-on-a-chip systems over chip-based tissue structures that are generated using the intrinsic cellular reorganization and self-assembly skills as demonstrated for or vascular, hepatic, cardiac as well as cerebral structures, the main question regarding the applications of bioprinting is now (*see* Figure 7): Where do we need precise spatial control over matrix densities, composition and cell types in a 3D tissue-like construct? The future of bioprinting lies, in the medium time, in the on-chip generation of architectural and biophysical cues by supporting and directing cell self-assembly and reorganization in a tissue-like construct with an initial template architecture. In other words, where ever microfluidic capabilities are the limiting factor, bioprinting can be used e.g. to locate different tissue structures in defined spaces on a common chip platform to guide the formation of mature multi-tissue structures such as i.e., the osteo-chondral unit, where elastic cartilage interfaces a very stiff calcified interface region before the subchondral bone structure. In many instances, microfluidic hydrogel loading is limited to certain range of stiffness, which do not represent the densities of any bone or cartilage tissue *in vivo*. Here, bioprinting can assist in generating a single cell-laden construct that features anisotropic elasticities, stiffness and composition gradients such as growth factors as well as degradable fibers. The precise directional control can also improve models interfacing with highly fibrous mechanically active tissues including meniscus, tendon and ligaments (Sun et al., 2017; Jian et al., 2021). Peridontal tissue engineering for example needs multi-cellular architectures and bone interfaces (Vurat et al., 2020). High resolution bioprinting using 2PP in particular could further generate stiffness gradients not only by proper material selection but also by tuning sub-micron hydrogel scaffold architectures using a variety of photosensitive bioinks (e.g. gelatin and even biocompatible ceramic composites) to fine tune anisotropic architectures. It is important to remember that architectural structures are linked to biomechanics and cellular mechanobiology, and thus can directly influence tissue physiology leading either to homeostasis or dysfunction as demonstrated for myogenic as well as tenogenic differentiation studies (Mondrinos et al., 2021). In other words, despite the current technological limitations and incompatibility, organ-on-a-chip technology and bioprinting are synergistic technologies that are ideally suited to create, in combination, the highest degree of microenvironmental control.

### Permission to Reuse and Copyright

Figures, tables, and images will be published under a Creative Commons CC-BY licence and permission must be obtained for use of copyrighted material from other sources (including re-published/adapted/modified/partial figures and images from the internet). It is the responsibility of the authors to acquire the licenses, to follow any citation instructions requested by third-party rights holders, and cover any supplementary charges.
